# Procedural sedation and analgesia in Swiss Pediatric Emergency Departments: a national subgroup analysis of a European cross-sectional survey

**DOI:** 10.1007/s00431-024-05701-5

**Published:** 2024-08-03

**Authors:** Fabrizio Romano, Gabriel Brändle, Olivia Abplanalp-Marti, Renato Gualtieri, Cyril Sahyoun

**Affiliations:** 1 Pediatric Emergency Department, Inselspital, University Hospital, University of Bern, Bern, Switzerland; 2 Division of Pediatric Emergency Medicine, Children’s Hospital of Geneva, Geneva University Hospitals, Rue Willy Donzé, 6, 1205 Geneva, Switzerland; 3 Division of Pediatric Emergency Medicine, Hirslanden Clinique Des Grangettes, Geneva, Switzerland; 4 Department of Pediatrics, Gynecology and Obstetrics, Geneva University, Geneva, Switzerland

**Keywords:** Procedural sedation, Analgesia, Switzerland, Pain, Pediatric Emergency Medicine

## Abstract

This study aims to provide a national overview of procedural sedation and analgesia practices within Pediatric Emergency Departments in Switzerland, focusing on the availability of pharmacologic agents, the presence of safety protocols, the utilization of non-pharmacological interventions, and to identify specific local limitations. We conducted a detailed subgroup analysis of Swiss data from a European cross-sectional survey on emergency department pediatric Procedural Sedation and Analgesia (PSA) practice, isolating data from Swiss sites. The survey, conducted between November 2019 and March 2020, covered various aspects of procedural sedation and analgesia practices. The survey included nine Swiss sites, treating a total of 252,786 patients in 2019. Topical analgesia, inhaled equimolar nitrous oxide-oxygen mixture, and ketamine were largely available. All sites had nurse-directed triage protocols in place; however, opioid administration was included in the protocols in only 66% of sites. Only 33% of hospitals reported common use of intravenous sedation. Barriers to procedural sedation and analgesia implementation included staffing shortages (89% of sites) and lack of dedicated spaces (78%).

*Conclusions*: Despite a broad array of pharmacological and options available in Swiss Pediatric Emergency Departments, challenges remain in standardizing practices across the country. Limited space and staffing and enhancing training on non-pharmacological interventions were identified as potential areas for improving pain and anxiety management in pediatric emergency care. This study underscores the need for national guidelines to harmonize emergency department PSA practices across Switzerland, ensuring all children have access to effective and evidence-based procedural comfort.

**Table:** 

**What is Known:** • *Recent research, conducted in European emergency departments, suggests that in pediatric Procedural Sedation and Analgesia (PSA) resources are limited, and practice is heterogeneous*
**What is New:** • *Swiss pediatric hospitals offer a wide range of pharmacological options for pain and anxiety management. However, significant barriers to PSA were identified. These include external control of intravenous sedation and insufficient integration of non-pharmacological interventions, such as child life specialists and procedural hypnosis. National guidelines are needed to harmonize PSA practices*

**What is Known:**

• *Recent research, conducted in European emergency departments, suggests that in pediatric Procedural Sedation and Analgesia (PSA) resources are limited, and practice is heterogeneous*

**What is New:**

• *Swiss pediatric hospitals offer a wide range of pharmacological options for pain and anxiety management. However, significant barriers to PSA were identified. These include external control of intravenous sedation and insufficient integration of non-pharmacological interventions, such as child life specialists and procedural hypnosis. National guidelines are needed to harmonize PSA practices*

## Introduction

Procedural sedation and analgesia (PSA) in pediatrics has evolved significantly over the past 20 years thanks to increased research, expanded practitioner competencies, access to more pharmacological agents, and growing awareness of the importance of pain and anxiety treatment during procedures [1]. Studies also highlight benefits such as improved patient flow and financial outcomes by avoiding costly solutions like general anesthesia or hospitalization [2].

A recent multi-national survey [3] however found numerous constraints and high heterogeneity in PSA among European emergency departments: although procedural sedation and analgesia is widely used in pediatric EDs, several challenges hinder its effective implementation. These include limited availability of certain medications, absence of standardized procedures, shortage of staff, insufficient space, and external control of sedation agents used in the ED.

The aim of this sub-analysis, focused on the Swiss data of the European survey, is to provide a nation-wide overview of the practice of PSA and to identify and address specific local limitations.

## Materials and methods

This study is a detailed subgroup analysis focused on the Swiss data from a cross-sectional European survey on pediatric emergency department PSA practice conducted between November 2019 and March 2020. Participants in the survey were the heads of EDs or their delegates responsible for PSA practices, each representing one site. They were contacted via email and invited to complete the questionnaire through a web link. The questionnaire covered various areas, such as the management of theoretical patients requiring PSA, the availability and frequency of use of medications, the characteristics of the personnel performing PSA and their training, existence of safety protocols, and barriers to the implementation of PSA. The distribution strategy employed a quota sampling method, aimed to involve an adequate number of facilities based on the population of each participating country. The methodology used has been further explained in the original study [3]. After obtaining approval from the original authors, Swiss sites participating in the survey were identified, and their data was isolated and analyzed. The potential for conducting sub-studies was included in the protocol of the main study, which was approved by the Swiss Association of Ethics Committees (2018–01889).

### Statistical analysis

Categorical data was presented as frequencies and percentages, continuous data as mean with 95% CI. Consistent with the original study, we reported the results as a proportion of the total number of children seen per year for patient-centered domains, and as a proportion of the total number of sites for site-centered domains. All analyses were conducted using Stata version 18 StataCorp. 2023 (Stata Statistical Software: Release 18. College Station, TX: StataCorp LLC). All associated graphs were produced using GraphPad Prism (version 10.0.0 for Windows, GraphPad Software, Boston, Massachusetts USA).

## Results

### Respondents

In the European survey, nine sites in Switzerland were invited to participate, all of which responded and were analyzed, representing a response rate of 100% (9/9) per the original study criteria. All surveyed sites were University Hospitals and/or Tertiary Care Centers and all sites cared for trauma patients. Among the 9 selected sites, 4 sites were in the German-speaking part of the country (out of 5 University Hospitals and/or Tertiary Care Centers in the area), 4 in the French-speaking part (out of 5 as well) and one in the Italian-speaking part (out of 1), ensuring a thorough and balanced representation of the entire nation.

In 2019, the mean number of children seen per year, per site, was 28,100 (95% CI 21,000–35,200), altogether representing a total of 252,786 patients.

### Management of a theoretical patient requiring PSA

When presented with the clinical situation of a 4-year-old child with a displaced forearm fracture requiring painful closed reduction and casting, all sites would perform the procedure using sedatives and analgesics. Most respondents (66%) preferred general anesthesia and treatment in the operating room. More than half of the respondents (55%) would consider nitrous oxide use, alone or combined with another analgesic and only 44% would consider intravenous (IV) deep or dissociative sedation in the ED. None of the sites none would treat the patient without sedatives or analgesics.

### Nurse-directed triage analgesia protocols, topical anesthetics, and minor trauma care

Nurse-directed triage analgesia protocols were in place at all sites, with all protocols including paracetamol and ibuprofen or similar non-steroidal anti-inflammatory drugs. Opioids available for nurse-directed triage administration were by oral route in 33% of hospitals, and intranasal fentanyl in 67%. Thirty-three percent of hospitals lacked opiates in nurse-directed triage analgesia protocols. All sites reported availability of topical anesthesia for both lacerations and for IV catheterization. Tissue adhesive for laceration repair was available in 89% (8/9) of the sites.

### Sedation and non-pharmacological care availability

Midazolam and nitrous oxide were available to all children seeking medical attention in all the hospitals surveyed. Other drugs that were widely available included ketamine (74% of children in 6/9 sites) and intranasal fentanyl (95% of children, 8/9 sites). Propofol (46% of children, 4/9 sites) and intranasal dexmedetomidine (13% of children, 1/9 site) were less commonly available.

Nitrous oxide/oxygen was used in equimolar concentration in all hospitals and one (18% of children) also offered higher concentrations, up to 70% N_2_O/30% O_2_.

Where available, the use of intravenous sedation, either with propofol or ketamine, was reported as uncommon (less than once a week) in 33% (3/9 hospitals), common (weekly or bi-weekly) in 33% (3 hospitals), and frequent (more than bi-weekly but less than daily) in 11%. No hospital reported daily use of IV sedation. Nitrous oxide was used at least daily in 89% of surveyed pediatric EDs (8/9).

Child life specialists and procedural hypnosis were available in two sites (22%). Only one site offered both approaches.

### Barriers to implementation of PSA

**Fig. 1:**
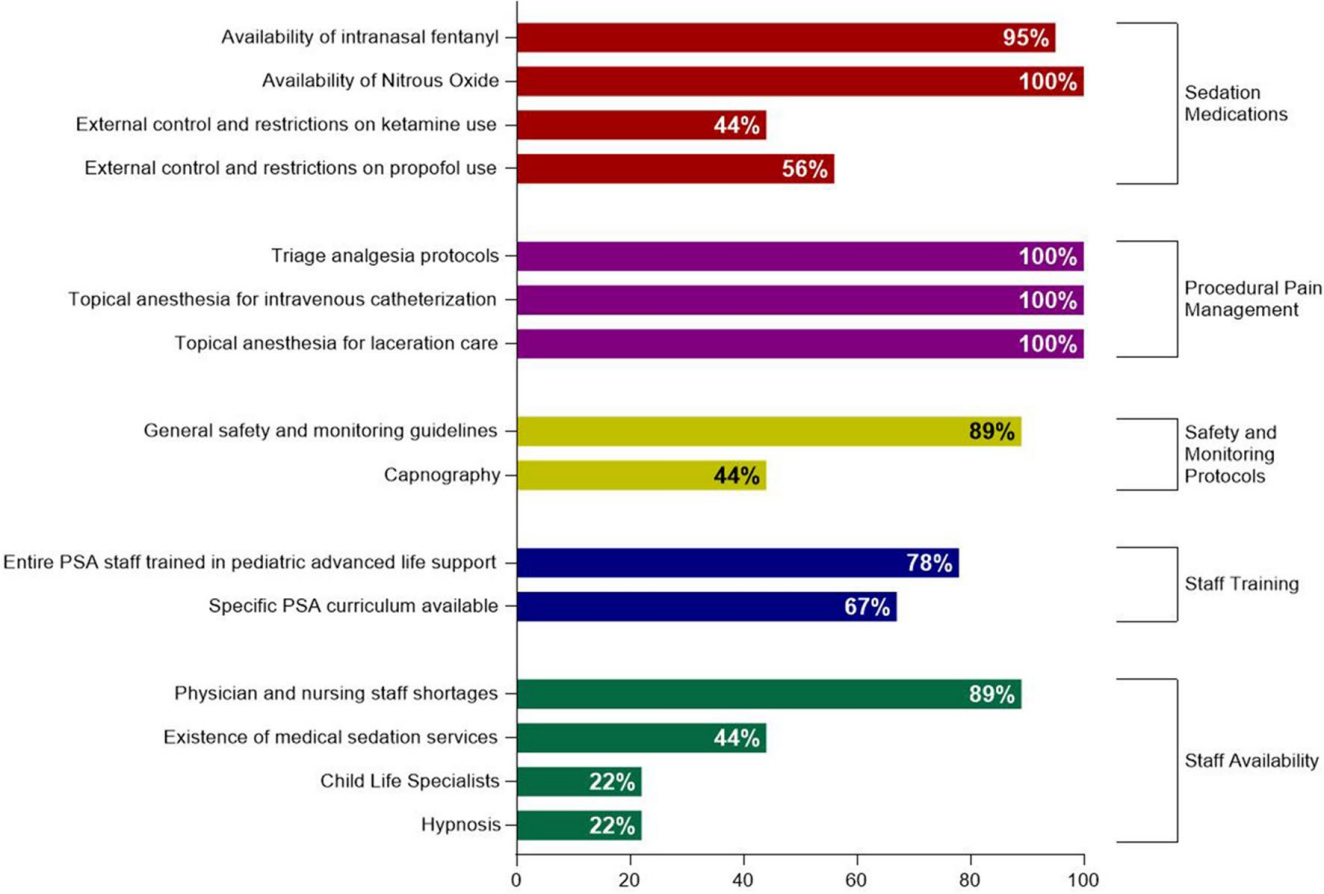
Prevalence of sedation and analgesia practices in Swiss pediatric emergency departments. PSA, procedural sedation analgesia; IV, intravenous; IN, intranasal

Shortages in physician and nursing staff who can respectively perform and monitor IV PSA was reported at 89% (8/9) of sites, and lack of a dedicated space was reported in 78% (7/9). Anesthesiologists reportedly controlled or restricted the use of ketamine and propofol in 44% (4/9) and 56% (5/9) of the sites, respectively. All survey respondents (9/9) agreed that ketamine was a useful agent for PSA in the ED. Results are summarized in Fig. 1.

## Discussion

Our results show that Swiss University Hospitals and Tertiary Care Centers Hospitals provide a wide range of pharmacologic options for pain and anxiety management in children during painful procedures. Staff training and PSA protocols are also widely available although there are still barriers to overcome. In the following sections, we discuss the adequate practices identified by this analysis and highlight the areas for further improvement.

### Adequate practices in place

When survey respondents were asked about the management of a theoretical patient requiring a painful procedure, the use of a combination of sedatives and/or analgesics was considered and available in 100% of the Swiss hospitals. This contrasts with the European study results [1] and with a similar sub-analysis of isolated Italian data [4] which revealed a lack of use of sedative/analgesic practices in 8% and 27% of the sites, respectively.

Furthermore, in all included centers, topical anesthesia was available for venous catheterization and laceration care, while nitrous oxide was widely used. All sites had nurse-directed triage analgesia protocols in place, allowing early management of acute pain by nursing staff from triage, a cost-effective way to improve pain management in the pediatric ED [5, 6]. Remarkably, intranasal fentanyl was available in nurse-directed triage analgesia protocols in most centers. As shown by previous implementation studies, protocols allowing access to intranasal fentanyl from triage further reduce the time to adequate analgesia and increase patient satisfaction [7, 8].

### Identifying areas for improvement

Our analysis has highlighted two critical areas necessitating improvement: the external control of intravenous sedation and the insufficient integration of non-pharmacological interventions.

- The fear of adverse events associated with intravenous sedation such as ketamine or propofol is a recurrent barrier in PSA implementation. Recent studies, however, have demonstrated that the risk is low in the hands of trained providers and when patients and pharmacological agents are critically selected [9–11]. With proper training, proper patient and drug selection, and using clear safety protocols, safe PSA can be practiced in the pediatric ED, by pediatricians.
- Crucially, the use of evidence based, non-pharmacological interventions such as specific training in pain and anxiety management of healthcare professionals, distraction and parental coaching, child life specialists, and procedural hypnosis, can be beneficial tools to reduce patient’s pain and distress in the pediatric ED [12, 13] . The identified barriers to further development such PSA modalities are mostly material. These consist largely of lack of staff availability, a shortage of trained staff, and the lack of a dedicated space in hospitals. As the patients’ rights movement gains momentum and evidence of the long-term impact of prior distressing episodes accumulates, the crucial role of multidisciplinary and multimodal pain management needs to be recognized and supported by every advocate and user of well conducted PSA programs [14]. Therefore, training on non-pharmacological interventions such as age-appropriate communication, guided imagery and hypnosis should be widely disseminated along with the understanding of the additional benefits provided by child life specialists and other comparable healthcare professionals.

### Limitations

Our study has several limitations. First, this is a retrospective subgroup analysis of self-reported practice, which makes human error in reporting and collecting the data a possibility. Another important issue is the inclusion of only University Hospitals and/or Tertiary Care Centers: our results are representative only of this type of hospital. Many regional hospitals also take care of minor procedures such as fracture reduction, primary burn care, or wound repair. PSA practices in regional hospital, which might be different than ones in University Hospitals and/or Tertiary Care Centers, are not captured in our data.

## Conclusion

Pediatric EDs in Switzerland offer a wide range of options related to PSA, including access to specific analgesics and sedatives and widespread nurse-directed triage analgesia protocols. However, there are several areas that require improvement, particularly the implementation of intravenous sedation when needed, and the enhancement of non-pharmacological options, such as child life specialists and hypnosis. This study serves as a foundation for harmonizing and implementing PSA practices across Switzerland to ensure a consistent, evidence-based, and effective approach to pain and anxiety management for all children seeking emergency care.

## Data Availability

Request for access to the data should be made to cyril.sahyoun@hug.ch. Data could be made available provided the applicant has appropriate ethics approval and approval from the authors of the original study, and a data transfer agreement is created.

## Abbreviations

CI: Confidence interval
ED: Emergency Department
IV: Intravenous
N2O: Nitrous oxide
O2: Oxygen
PSA: Procedural sedation and analgesia
